# Safety risks among frail older people living at home in the Netherlands – A cross‐sectional study in a routine primary care sample

**DOI:** 10.1111/hsc.13230

**Published:** 2020-11-17

**Authors:** Manon Lette, Annerieke Stoop, Giel Nijpels, Caroline Baan, Simone de Bruin, Hein van Hout

**Affiliations:** ^1^ Department of General Practice and Elderly Care Medicine Amsterdam Public Health Research Institute Amsterdam University Medical Centres – VU University Amsterdam The Netherlands; ^2^ National Institute for Public Health and the Environment Bilthoven The Netherlands; ^3^ Scientific Centre for Transformation in Care and Welfare (Tranzo) University of Tilburg Tilburg The Netherlands; ^4^ SIGRA Amsterdam Thee Netherlands

**Keywords:** care of elderly people, health needs assessment, multi‐disciplinary, primary care, risk assessment, risk in community care

## Abstract

Frail older people face a range of problems and risks that could undermine their ability to live safely at home. A comprehensive overview of these risks, from a multidimensional perspective, is currently lacking. This study aims to examine the prevalence of risks in multiple domains of life among frail older people living at home. We used cross‐sectional data from 824 people aged 65 years and older, who received a comprehensive geriatric assessment (the interRAI Home Care [interRAI‐HC]) between 2014 and 2018, as part of routine care from 25 general practices in the region of West‐Friesland, the Netherlands. The interRAI‐HC identifies amenable risks related to people's clinical conditions, functioning, lifestyle and behaviour, and social and physical environment. Descriptive statistics were used to examine population characteristics (age, gender, marital status, living arrangements and presence of chronic conditions) and prevalence of risks. Most common risks were related to people's clinical conditions (i.e cardio‐respiratory health, urinary incontinence, pain), functioning (i.e. limitations in instrumental activities of daily living and mood) and social environment (i.e. limitations in informal care and social functioning). More than 80% of frail older people faced multiple risks, and often on multiple domains of life simultaneously. People experiencing multiple risks per person, and on multiple domains simultaneously, were more often widowed and living alone. The multidimensional character of risks among frail older people living at home implies that an integrated approach to care, comprising both health and social care, is necessary. Insight in the prevalence of these risks can give direction to care allocation decisions.

**What is known about this topic?**

- Frail older people's ability to live safely at home may be undermined by a range of problems and risks on multiple domains of life.
- Combinations of problems and risks on multiple life domains have been shown to be associated with poorer health outcomes.
- A comprehensive overview of the prevalence of risks across multiple domains of life is currently lacking.

**What this paper adds?**

- The majority of frail older people living at home face problems and risks in multiple domains of life simultaneously.
- Risks related to clinical conditions, functional limitations, and social environment were most prevalent.
- These insights can give further direction to the allocation of integrated care and support in the community.

## 1 BACKGROUND

Many people live independently at home until old age (World Health Organization, 2015). As people age, they may become frail and experience problems and needs that could pose risks to their ability to live safely at home (Marengoni et al., 2011; Van Blijswijk et al., 2015; World Health Organization, 2015). These risks can occur in multiple domains of older people's lives (Abdi et al., 2019). For example, older people may face risks related to their health and functioning, such as physical or cognitive decline, or to their lifestyle and behaviour, such as unhealthy habits or poor self‐care. Furthermore, they could encounter risks related to their social or physical environments, such as social isolation, caregiver burden or hazards in the home (Lang et al., 2008; Lau et al., 2007; Vincent & Amalberti, 2016).

The multidimensional nature of risks to which older people are exposed has been widely recognised (Abdi et al., 2019; De Donder et al., 2019). Over the years, studies have examined a broad array of risks that could undermine people's ability to live safely at home, and their association with outcomes such as adverse health consequences, hospitalisation and institutionalisation (e.g. Dent & Hoogendijk, 2014; Doran et al., 2009; Gill et al., 2005; Kuzuya et al., 2011; Leendertse et al., 2008). However, up until now research has mostly focused on isolated risks or specific combinations of risks. Studies reporting on the range of risks that occur among frail older people, from a multidimensional perspective, are scarce. Yet such insights are important, especially since combinations of problems on multiple domains of life have been shown to be associated with poorer health outcomes (Van Houwelingen et al., 2015).

In daily clinical practice, insight into the risks that occur among a frail older person may be gained through the use of a comprehensive geriatric assessment (CGA). Health and social care providers may use a CGA to identify amenable risks on multiple life domains, and subsequently develop care plans in which strategies to manage risks are established (Stoop et al., 2019; van Rijn et al., 2016). In addition to their clinical purpose, the results of such assessments also provide the opportunity to assemble a comprehensive overview of the range of risks that occur among a population of frail older people. Such an overview generates knowledge on the type of care and support that this population requires, which may inform policy makers and practitioners in decisions on resource allocation.

We aim to contribute to a better understanding of the range of amenable risks that are prevalent among frail older people living at home, using a multidimensional perspective. Using data collected in a primary care setting by means of a CGA that is part of routine care for older people, this study addresses the following research question: what is the prevalence of risks across multiple domains of life among frail older people living at home?

## 2 METHODS

### 2.1 Setting and study population

This cross‐sectional study used deidentified data from the interRAI database stored at Amsterdam University Medical Centres – location VU University. This database contains information generated by the interRAI Home Care (interRAI‐HC) (Morris et al., 2009), which is a standardised and fully structured CGA instrument. The interRAI‐HC is used as part of routine care in 25 general practices in a rural urbanised area in the province of North‐Holland, the Netherlands. In the Netherlands, everyone is registered within a general practice. The general practices working with the interRAI‐HC apply a case finding approach to identify frail older people, after which practice nurses conduct home‐based assessments. The case finding approach is based on the General Practitioners’ (GP) clinical judgement and was previously found to identify frail older persons in a valid way (Hoogendijk et al., 2012; Sutorius et al., 2016). Outcomes of the interRAI‐HC assessments are used by GPs to further inform the care planning process. The study population for this study consisted of frail older people (aged ≥65 years) living at home, who received their first interRAI‐HC assessment from their general practice between 2014 and 2018.

### 2.2 Data collection

Data were collected at older people's homes by trained practice nurses, who used an application of the interRAI‐HC instrument. The interRAI‐HC, which was developed by the interRAI network (www.interrai.org), contains approximately 300 items that cover a person's demographics, physical and cognitive functioning, psychosocial and emotional well‐being, living environment and medical diagnoses and conditions. Items in the interRAI‐HC have shown substantial reliability (Hirdes et al., 2008) and the instrument provides the opportunity to integrate information from direct observation, medical records and communication with the person under assessment and their informal care network. Furthermore, the interRAI‐HC includes a series of validated Clinical Assessment Protocols (CAPs; Morris et al., 2010). CAPs are triggered by algorithms embedded in the software supporting interRAI instruments. CAPs alert the assessor to specific problems that can be addressed in care planning. A key characteristic is that they trigger only if there is a possibility to intervene (i.e. a problem could either be averted, a person's circumstance could be improved or further deterioration could be prevented). As such, a triggered CAP can be considered a ‘red flag’ marking an amenable health risk.

### 2.3 Outcome measures

The outcome measures included the risks, which were defined as 22 CAPs. CAPs were recoded into dichotomised variables (i.e. presence or absence of trigger). Table 1 provides a short description of each CAP, which we categorised into the following risks categories, reflecting different domains of people's lives: (a) clinical status, (b) daily functioning, (c) lifestyle and behaviour, and (d) social and physical environment. Detailed information on the CAPs and their underlying algorithms is available elsewhere (Morris et al., 2010). However, it should be noted that CAPs are created based on multiple underlying variables, which means they are recorded as missing whenever any one of the underlying variables is missing.

**TABLE 1:** Description of risks identified through Client Assessment Protocols (CAPs) in the interRAI Home Care assessments instrument (Morris et al., 2010)

Risk category	CAP	Description
Clinical status	Cardio‐respiratory health	Identifies people who suffer from cardio‐respiratory conditions (e.g. chest pain, shortness of breath, irregular pulse, dizziness)
	Dehydration	Identifies people who show signs of dehydration or a disrupted fluid balance
	Delirium	Identifies people who show active symptoms of delirium (e.g., easily distracted, unstable consciousness, acute cognitive decline)
	Faecal incontinence	Identifies people who suffer from faecal incontinence, and for whom bowel function could be improved or decline could be prevented
	Nutrition	Identifies people who show signs of malnutrition
	Pain	Identifies people who suffer from pain on a daily basis
	Pressure ulcer	Identifies people with pressure ulcers or people who are at risk of developing pressure ulcers
	Urinary incontinence	Identifies people who suffer from urinary incontinence, and for whom bladder function could be improved or decline could be prevented
Functioning	ADL	Identifies people who are at risk of a decreasing ability to independently perform ADL or for whom ADL abilities could be improved. ADL include basic self‐care tasks such as walking, feeding, bathing, dressing, grooming, toileting and transferring
	Cognitive functioning	Identifies people with no or only mild cognitive impairments, who show at least two risk factors for cognitive decline (e.g., dementia, communicative problems, disorientation, confusion, restlessness)
	Communication	Identifies people with communicative problems (i.e., problems with expressing themselves and/or understanding others) that could be improved, or for whom further decline could be prevented
	Falls	Identifies people who experienced one or more fall incidents during the past 90 days, who are at risk of experiencing another fall incident
	IADL	Identifies people for whom IADL ability could be improved and who have no or only mild cognitive impairments. IADL include self‐care tasks that require more complex thinking skills, such as shopping, meal preparation, home maintenance and managing finances, communication, transportation and medications
	Mood	Identifies people who are at risk of developing a depressive disorder
	Risk of institutionalisation	Identifies people who are at high risk of admittance to an institutional care facility in the following months
Lifestyle and behaviour	Behaviour	Identifies people who have shown behavioural problems (e.g., wandering, verbal or physical violence, socially inappropriate behaviour) during the last 3 days
	Physical activity	Identifies people with <2 hr of physical activity in the last 3 days, who do not have (physical) limitations to be more physically active
	Smoking and drinking	Identifies people who smoke on a daily basis and consume alcohol on an incidental to regular basis
Social and physical environment	Abusive relationship	Identifies people who are at risk of abuse, based on one or more indicators of abuse (e.g., scared of relative or caregiver, showing signs of neglect or maltreatment) combined with one or more stress factors (e.g., BMI < 18, depression, social isolation, upset caregiver)
	Home environment	Identifies people who show at least two signs of frailty (e.g., unable to climb stairs, unstable gait, poor or unstable health, depressive symptoms, hallucinations) and live in a problematic home environment (e.g. dilapidation, filth, problems with lighting, carpets, kitchen, bathroom, access to rooms)
	Informal care	Identifies people who need help with at least one IADL area and who have a brittle informal support network (i.e., at least two of the following: spend most of their time alone, live alone, have no primary informal caregiver)
	Social function	Identifies people who report feeling lonely or who show no or declined social involvement in their community

Abbreviations: ADL, activities of daily living; BMI, body mass index; IADL, instrumental activities of daily living.

### 2.4 Background characteristics

Background characteristics of older people included age, gender, marital status, living arrangements, and the presence of a number of chronic conditions (cancer; congestive heart failure [CHF], coronary heart disease; chronic obstructive pulmonary disorder; dementia; diabetes; stroke). Although the interRAI‐HC provides information on a wider range of diagnosed conditions, we chose to include only those conditions that occurred in at least 10% of the sample.

### 2.5 Data analysis

Analyses were performed using SPSS Version 24. Descriptive statistics were applied to examine the characteristics of the study population, the prevalence of triggers for each CAP, the number of triggered CAPs per person and the number of triggered risk categories per person. A risk category was triggered when there was a trigger present for at least one CAP in that risk category. Using cross‐tabulations, the percentage of triggered CAPs across different demographic subgroups in the sample was examined. Pearson Chi‐Square tests (for alpha *p* < 0.001) were used to determine significant relationships between CAPs and demographic variables.

### 2.6 Ethics statement

Assessments were performed for clinical purposes as part of routine care. After de‐identification, data were transferred to the interRAI database at the Amsterdam University Medical Centres – location VU University. An opt‐out procedure was applied in compliance with the EU General Data Protection Regulation. Older people were informed in general terms that their data could be used for research purposes by their practice nurses and through their practices’ newsletters, websites and posters in waiting rooms, and they had the possibility to object.

## 3 RESULTS

**FIGURE 1:**
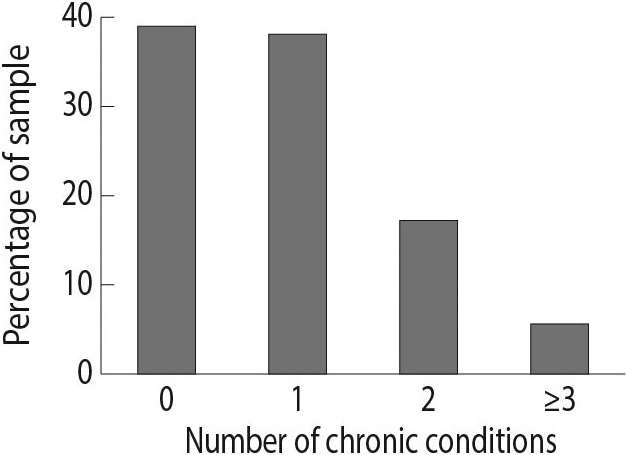
Number of chronic conditions per person among people who received an interRAI Home Care assessment in primary care practices in West‐Friesland, the Netherlands (*N* = 824). Chronic conditions include cancer, congestive heart failure, coronary heart disease, chronic obstructive pulmonary disorder, dementia, diabetes and stroke

A total of 824 cases were included in the study sample. Table 2 summarises the characteristics of the sample. Age ranged from 65 to 100 years, with a mean age of 83.4 years. One‐third of the sample was male. As shown in Figure 1, almost 61% of the sample had at least one medical diagnosis, of which diabetes was the most prevalent.

**TABLE 2:** Characteristics of frail older people who received an interRAI Home Care assessment in primary care practices in West‐Friesland, the Netherlands, between 2014 and 2018 (*N* = 824)

	*n* (%)	*N*	% missing
Age		824	0.0
65–74	55 (6.7)		
75–84	403 (48.9)		
85+	366 (44.4)		
Gender		787	4.5
Male	255 (30.9)		
Female	532 (64.6)		
Marital status		671	18.6
Married/in relationship	236 (28.6)		
Widowed	379 (46.0)		
Divorced	19 (2.3)		
Never married	37 (4.5)		
Living arrangements		801	2.8
Alone	540 (65.5)		
With partner	237 (28.8)		
With others	24 (2.9)		
Diagnoses			
Cancer	84 (10.2)	746	9.5
CHF	118 (14.3)	750	9.0
CHD	101 (12.3)	750	9.0
COPD	88 (10.7)	749	9.1
Dementia	80 (9.7)	750	9.0
Diabetes	190 (23.1)	757	8.1
Stroke	93 (11.3)	752	8.7

Note

*n* = number of cases in the specific subsample; *N* = total number of valid cases.

Abbreviations: CHD, coronary heart disease; CHF, congestive heart failure; COPD, chronic obstructive pulmonary disorder.

**FIGURE 2:**
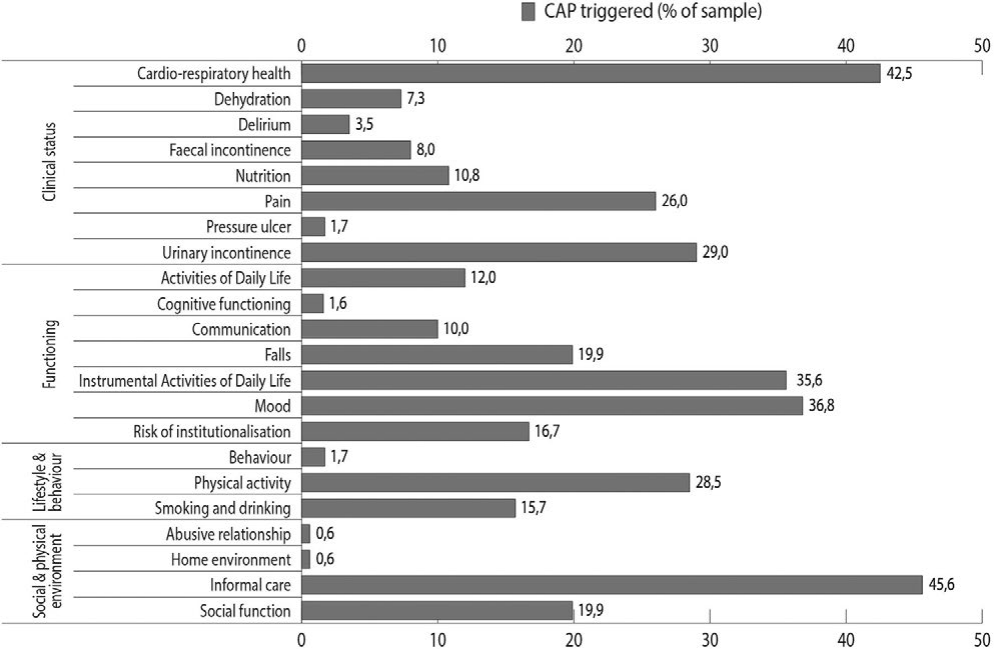
Prevalence of triggered CAPs among older people who received an interRAI Home Care assessment in primary care practices in West‐Friesland, the Netherlands (*N* = 824). CAPs stand for Client Assessment Protocols, which are validated algorithms that alert the assessor to specific problems and risks that can be addressed

**FIGURE 3:**
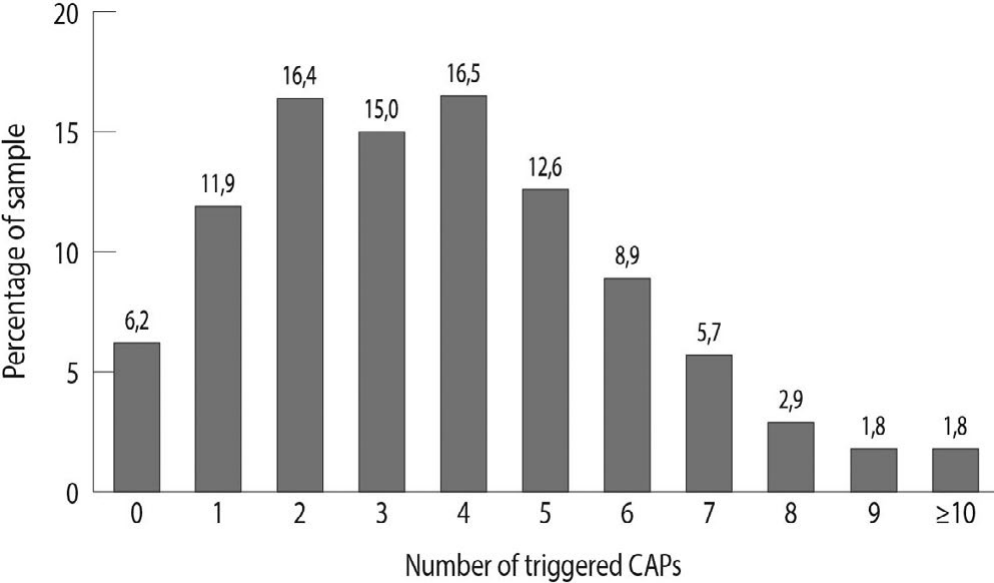
Number of triggered CAPs per person among people who received an interRAI Home Care assessment in primary care practices in West‐Friesland, the Netherlands (*N* = 824, total number of CAPs = 22). CAPs stand for Client Assessment Protocols, which are validated algorithms that alert the assessor to specific problems and risks that can be addressed

**FIGURE 4:**
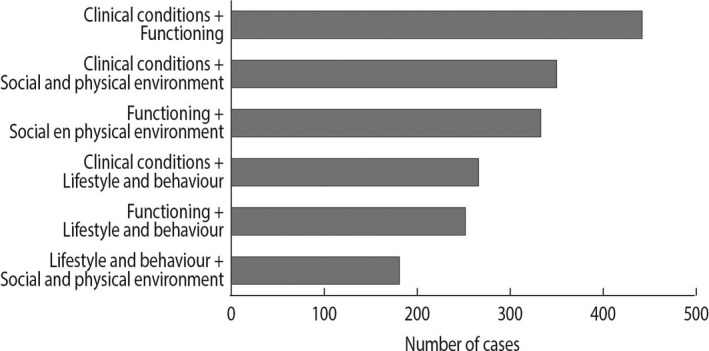
Combinations of triggered risk categories among people who received an interRAI Home Care assessment in primary care practices in West‐Friesland, the Netherlands (*N* = 824). A risk category was triggered when one or more Client Assessment Protocols in that category presented a trigger

Figure 2 presents the prevalence of triggered CAPs. CAPs for cardio‐respiratory health, informal care, mood and instrumental activities of daily living (IADL) were triggered most often. As shown in Figure 3, the number of triggered CAPs per person ranged from zero to thirteen, with a median of four triggered CAPs per person. Approximately 15% of the sample triggered CAPs in one risk category only, whereas approximately 29%, 31% and 16% of the sample triggered CAPs in, respectively, two, three or four risk categories simultaneously. In those cases where multiple risk categories were triggered simultaneously, the most common combinations of categories were (a) clinical conditions and functioning, (b) clinical conditions and social environment, and (c) functioning and social environment, as shown in Figure 4. People who presented multiple triggered CAPs per person, or triggered CAPs in multiple risk categories simultaneously, were more often widowed, living alone and diagnosed with at least one chronic condition (see additional Figures in Appendix S1).

Appendix S2 shows additional tables providing the prevalence of triggered CAPs stratified by age, gender, marital status, living arrangements and the presence or absence of a number of chronic conditions. Triggered CAPs for urinary incontinence and pain were more often observed among women than men. Risk of malnutrition was observed especially in people aged ≥85 years and people who lived alone. IADL was triggered more often in people aged ≥85 years, whereas mood was triggered more often in the younger age groups. Almost 20% of the sample was at risk of experiencing a fall incident, more often in men than women. Almost 30% of the sample had amenable low levels of physical activity, more often among people with CHF. The CAP for informal care was triggered only among people who lived alone, and more often among women than men and among people aged ≥85 years. The CAP for social functioning was triggered more often among people who lived alone.

## 4 DISCUSSION

This study demonstrated that the prevalence of risks related to clinical conditions and symptoms was high among older people living at home. It also showed a high prevalence of risks related to functional impairments and limited social support. Furthermore, we found that the majority of older people faced multiple risks, and often in multiple domains of life simultaneously. This observation is important, since a higher number of life domains in which problems are observed is associated with an increase in adverse health outcomes (Van Houwelingen et al., 2015), and as such, may undermine older people's ability to live safely at home.

To our knowledge, this is one of the first studies to provide a comprehensive overview of the range of risks present among this population from a multidimensional perspective. Although previous findings showed that problems and risks in the clinical, functional, behavioural and social domains of life are common among frail older people living at home (Hoogendijk et al., 2014; van Rijn et al., 2016), our study adds to the literature in showing that combinations of risks across these multiple domains of life are highly prevalent. Current literature suggests that many problems and risks are, indeed, interrelated. For example, the association between multimorbidity and depressive disorders has been well‐established (Read et al., 2017), and both risks are also associated with functional decline (Stuck et al., 1999). Limitations in the physical, cognitive and psychological domains can explain insufficient physical activity (Gomes et al., 2017), and in turn, physical inactivity is associated with limitations in activities of daily living (Tak et al., 2013). Furthermore, physical limitations, such as urinary incontinence, are associated with social isolation among older people (Nicholson, 2012). The co‐occurrence and interrelatedness of problems and risks confirms the urgency to address older people's safety at home in a multidimensional way.

In contrast with previous findings (Carter et al., 1997), we found a low prevalence of risks related to people's home environments. An explanation for this might be that in the interRAI‐HC instrument, the risk related to people's home environment mainly included issues such as dilapidation, problems with lighting and room access. In the Netherlands, however, risks related to people's home environment stem primarily from barriers related to home maintenance or people's ability to finance home adaptations (Hoogendijk et al., 2014; Lette et al., 2017). These items were not included in the interRAI‐HC, and as such, the risk for home environment as defined in this study may therefore not be entirely relevant to the Dutch context.

### 4.1 Study limitations

Several limitations should be considered. First, GPs used a case finding approach to select frail older people for assessment. The exact approach used varied across different practices, depending on the GPs’ preferred way of working. Generally, however, the selection for CGA with interRAI‐HC was based on GPs’ clinical judgement of people's frailty status, rather than on a standardised and validated frailty identification tool. The lack of standardised information on people's frailty status means we have no information about the generalisability of our findings to the general population of frail older people living at home. However, GPs’ judgement of frailty has previously been shown to result in a valid and representative selection of frail older people in primary care (Hoogendijk et al., 2012; Sutorius et al., 2016). Furthermore, a national report on frail older people in the Netherlands indicates that frail older people living at home are often older, female and living alone (Van Campen, 2011), which resembles the demographic profile of this study's population of frail older people living at home.

Second, the lack of data on medication use is a limitation, especially since other studies suggest that problems with medication are prevalent among older people living at home (Hoogendijk et al., 2014; van Rijn et al., 2016). Although a CAP flagging medication‐related risks is included in the interRAI‐HC, the underlying variables were not available in our sample because medication‐related information was collected and monitored by the local pharmacies, rather than by the general practices. Third, our data only provides insight into the prevalence of risks that are amenable, since CAPs trigger only when intervention is possible. Therefore, in some cases our findings may reflect an underestimation of the risk prevalence. For example, CAPs such as (I)ADL, cognitive functioning or incontinence will not trigger in case of severe cognitive impairment, as this is considered to limit the possibility for intervention. However, in this study, severe cognitive impairment was only present in approximately 1.5% of the sample.

Another limitation is that information on whether or not a CAP was triggered was not always available. The percentage of these missing CAPs varied per CAP, with a median percentage of 8.85% (range 2.4%–50.2%). Missing CAPs resulted from missing values in the CAPs’ underlying variables. Missing values are inevitable, especially since we used data from an instrument used in daily clinical practice. Higher rates of missing values were found for items that required specific action from the practice nurse carrying out the assessment, for example the measurement of height and weight. In a minority of cases, missing values were the result of selective completion of the assessment. Both types of missing values could lead to an underestimation of the prevalence of risks found in this study. More accuracy in the completion of assessments could further improve the reliability of the interRAI‐HC.

### 4.2 Implications

This study showed that the use of interRAI‐HC in routine practice supported general practices to identify a wide range of risks among the frail older people in their care population. These insights serve clinical purposes on individual level, since CAPs support care planning processes by equipping care providers with evidence‐informed guidelines for treatment and support. On population level, the insights gained from this study can give direction to the approach to care for older people living at home.

Our finding that risks often occurred concurrently, and in multiple domains of life simultaneously, confirms that an integrated approach to care and support is necessary (Van Houwelingen et al., 2015). Indeed, care commissioners and service providers are increasingly adopting transformation towards integrated care (World Health Organization, 2015, 2016). Organising services in a way that they are person‐centred, proactive and coordinated across different providers of care and support is expected to contribute to higher quality care and support, that is safe, timely and respectful of people's individual preferences (de Bruin et al., 2018; Institute of Medicine Committee on Quality of Health Care in America, 2001; World Health Organization, 2016). In addition to these efforts, our findings suggest that acknowledging the interrelatedness of risks is especially important when organising care and support for older people living at home. Addressing risks in a way that acknowledges this interrelatedness requires an interdisciplinary team of care providers, who understand how multiple risks can accumulate to undermine older people's safety and who are able collaborate beyond their individual areas of expertise.

The high prevalence of risks related to domains beyond older people's clinical status, such as IADL and psychosocial functioning, further suggest that a comprehensive perspective on care and support for older people living at home is necessary. Traditionally, care for older people living at home has focused primarily on the clinical implications of ageing, such as frailty, multimorbidity and physical limitations (Fried et al., 2004; Hébert, 1997; Hoogendijk et al., 2019; Marengoni et al., 2011; Ryan et al., 2015). However, broader concepts of health are increasingly being adopted. These concepts view ageing as part of life and include aspects such as functioning, resilience, well‐being and quality of life (Hendrikx et al., 2019; Huber et al., 2011; Lemmens et al., 2019; Vree et al., 2018). In line with this shifting perspective, our findings suggest that efforts should be taken to combine medical and non‐medical solutions in integrated care for older people living at home. Collaboration between health and social care providers, as well as support from community resources, seem critical in order to support older people to live safely at home. Taking these observations into account in resource allocation decisions could further support efforts towards high quality care and support.

## 5 CONCLUSION

Routinely collected data from clinical practice can provide important insights that could direct decisions on the allocation of care and support for frail older people living at home. This study shows that most frail older people face multiple risks, and on multiple domains of life simultaneously. This confirms that a comprehensive approach to care is necessary, in which special attention should be paid to integrating medical, practical and social care and support.

## CONFLICT OF INTEREST

The authors declare that they have no competing interests.

## AUTHORS' CONTRIBUTIONS

All authors were involved in the conception and design of the paper. HvH was involved in data acquisition, and ML analysed the data with support from HvH and GN. All authors together interpreted the data. ML drafted the manuscript an AS, GN, CB, SdB and HvH critically revised the manuscript. All authors approved of the final manuscript.

## Supporting information

Appendix S1

Appendix S2

## Data Availability

The data supporting the findings of this study are available upon reasonable request by contacting prof.dr. Hein van Hout (email: hpj.vanhout@amsterdamumc.nl).
