# Supplementary material for: Safety risks among frail older people living at home in the Netherlands – A cross‐sectional study in a routine primary care sample
Source: Health Soc Care Community. 2020 Nov 17;30(2):e469–77. doi: 10.1111/hsc.13230 (PMC9292903; doi:10.1111/hsc.13230)
Supplement: Supplementary file 1 — Appendix S1 [file HSC-30-e469-s001.pdf]

Supplementary file 1

Paper Title: Safety risks among frail older people living at home in the Netherlands - a cross-sectional study in a routine primary care sample

Description: Additional Figures

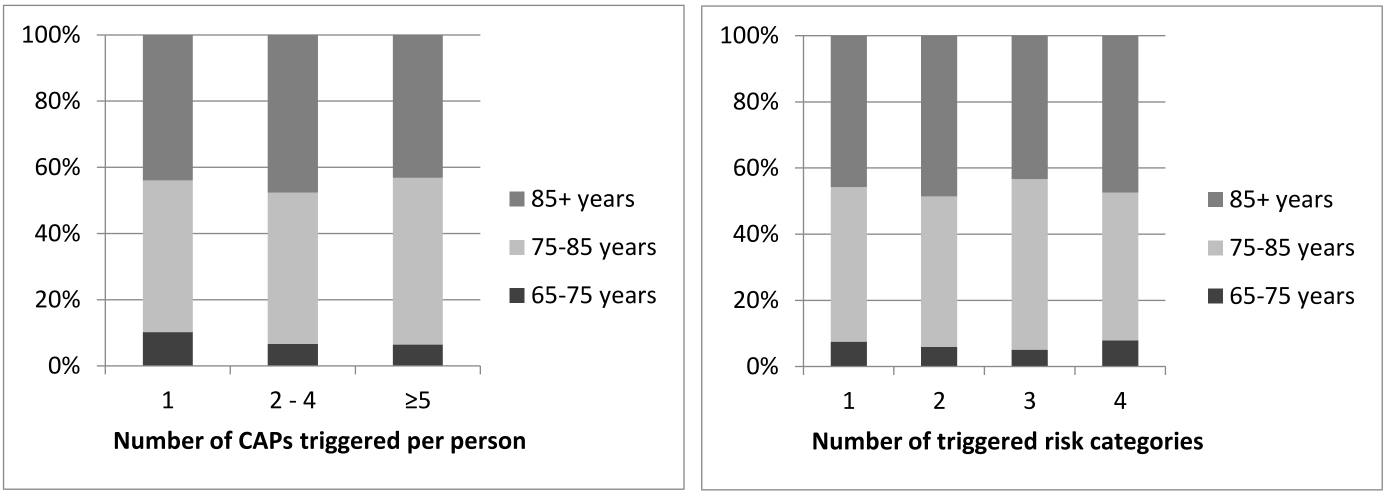

Figure A1. Number of triggered CAPs per person, and number of triggered risk categories, stratified by age. CAPs stand for Client Assessment Protocols, which are validated algorithms that alert the assessor to specific problems and risks that can be addressed. A risk category was triggered when one or more CAPs in that category presented a trigger.

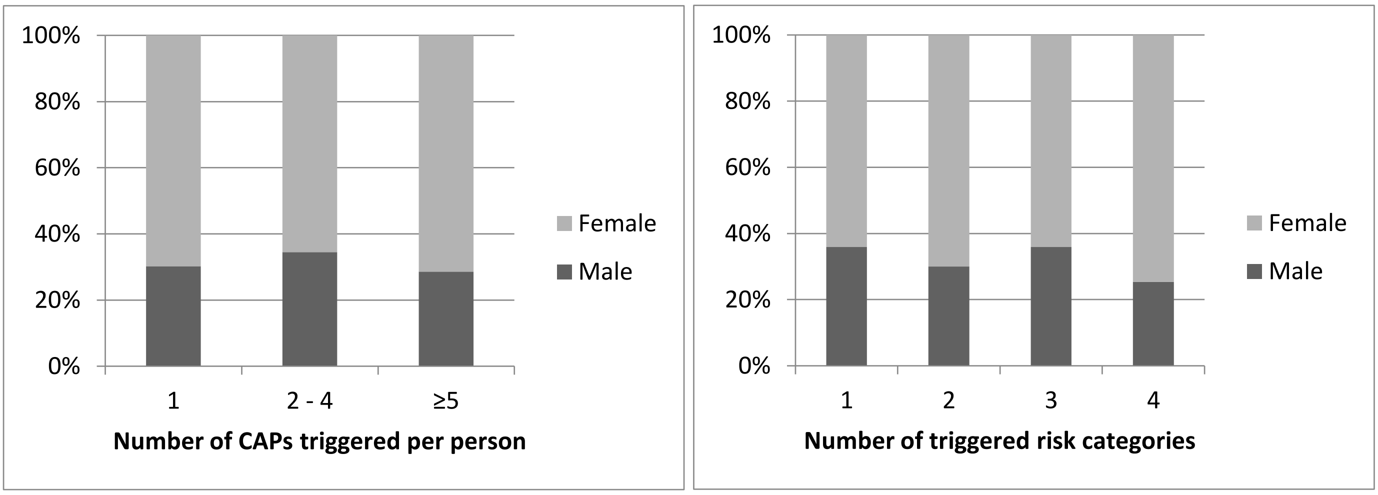

Figure A2. Number of triggered CAPs per person, and number of triggered risk categories, stratified by sex. CAPs stand for Client Assessment Protocols, which are validated algorithms that alert the

assessor to specific problems and risks that can be addressed. A risk category was triggered when one or more CAPs in that category presented a trigger.

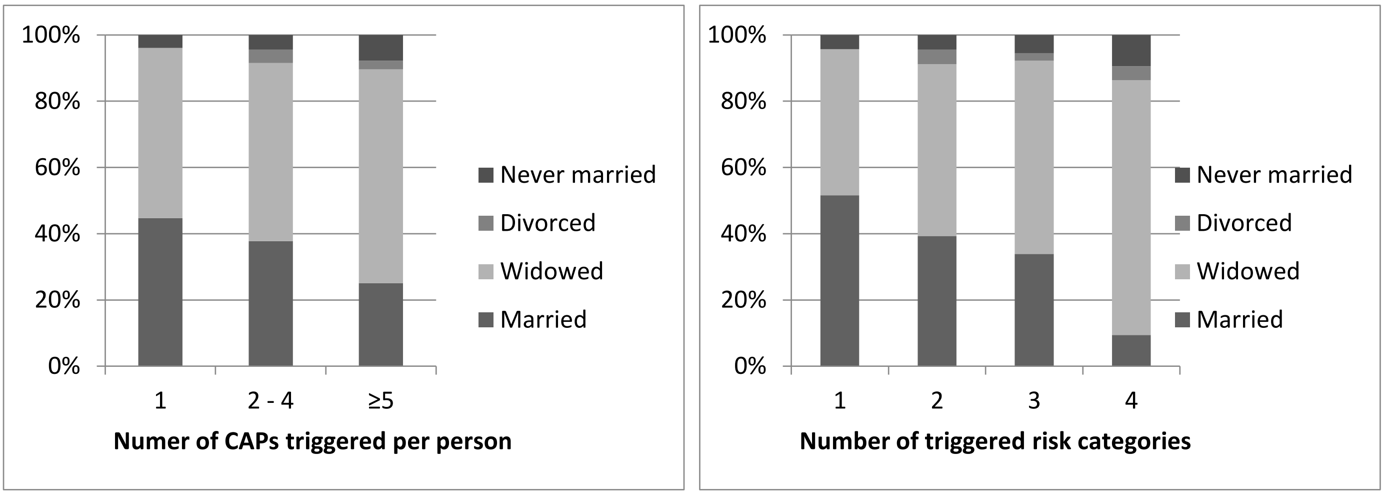

Figure A3. Number of triggered CAPs per person, and number of triggered risk categories, stratified by marital status. CAPs stand for Client Assessment Protocols, which are validated algorithms that alert the assessor to specific problems and risks that can be addressed. A risk category was triggered when one or more CAPs in that category presented a trigger.

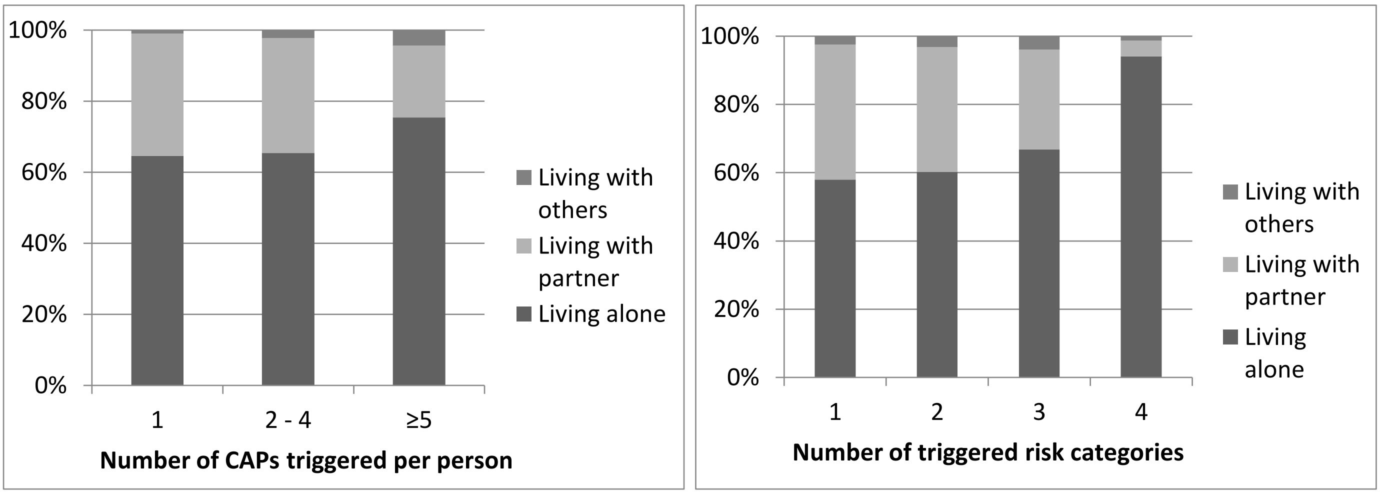

Figure A4. Number of triggered CAPs per person, and number of triggered risk categories, stratified by living arrangements. CAPs stand for Client Assessment Protocols, which are validated algorithms that alert the assessor to specific problems and risks that can be addressed. A risk category was triggered when one or more CAPs in that category presented a trigger.

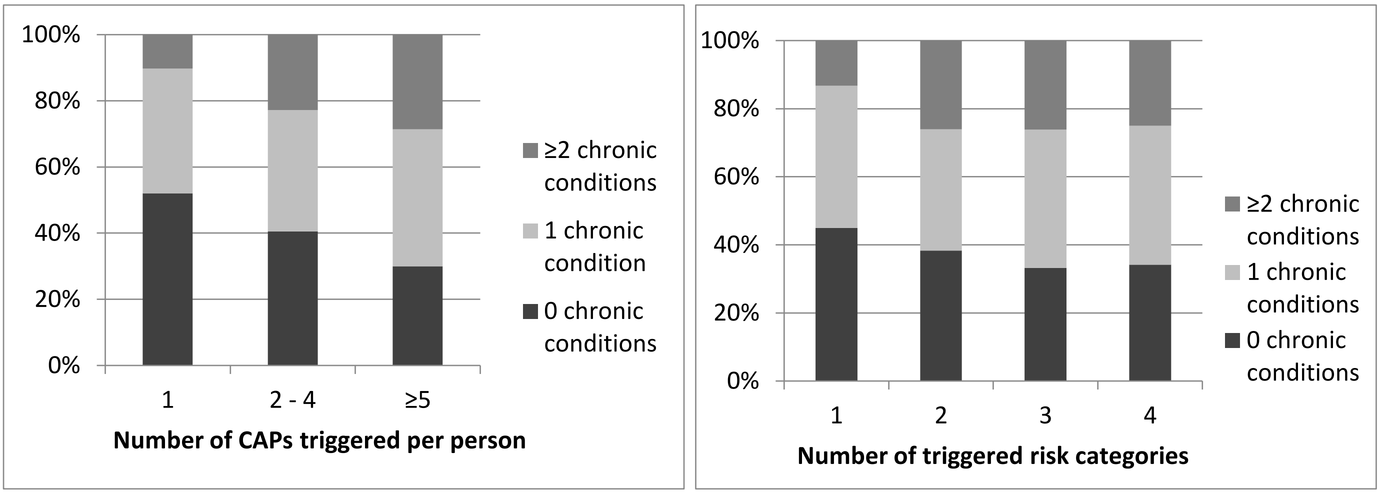

Figure A5. Number of triggered CAPs per person, and number of triggered risk categories, stratified by number of chronic conditions. CAPs stand for Client Assessment Protocols, which are validated algorithms that alert the assessor to specific problems and risks that can be addressed. A risk category was triggered when one or more CAPs in that category presented a trigger.
