# Supplementary material for: Safety risks among frail older people living at home in the Netherlands – A cross‐sectional study in a routine primary care sample
Source: Health Soc Care Community. 2020 Nov 17;30(2):e469–77. doi: 10.1111/hsc.13230 (PMC9292903; doi:10.1111/hsc.13230)
Supplement: Supplementary file 2 — Appendix S2 [file HSC-30-e469-s002.pdf]

Supplementary file 2

Paper title: Safety risks among frail older people living at home in the Netherlands - a cross-sectional study in a routine primary care sample

Description: Additional Tables

See next pages

Table A1. Absolute number and prevalence of triggered CAPs related to 'Clinical status', in total sample and stratified in subgroups (legend below the table).

	**CAPs[†] related to clinical status**															
	**Cardio-respiratory conditions**		**Dehydration**		**Delirium**		**Faecal incontinence**		**Nutrition**		**Pain**		**Pressure ulcer**		**Urinary incontinence**	
	**n (%)**	**N**	**n (%)**	**N**	**n (%)**	**N**	**n (%)**	**N**	**n (%)**	**N**	**n (%)**	**N**	**n (%)**	**N**	**n (%)**	**N**
**Total sample**	**350 (42.5)**	**794**	**60 (7.3)**	**775**	**29 (3.5)**	**793**	**66 (8.0)**	**701**	**89 (10.8)**	**591**	**214 (26.0)**	**766**	**14 (1.7)**	**651**	**239 (29.0)**	**725**
Age																
65-74	15 (30.6)	49	3 (6.0)	50	4 (7.7)	52	5 (12.2)	41	2 (5.6)	36	13 (27.1)	48	3 (7.9)	38	16 (39.0)	41
75-84	177 (45.4)	390	30 (7.9)	378	15 (3.9)	386	22 (6.3)	347	36 (11.6)	311	129 (33.7)	383	9 (2.8)	320	107 (29.7)	360
85+	158 (44.5)	355	27 (7.8)	347	10 (2.8)	355	39 (12.5)	313	51 (20.9)	244	72 (21.5)	335	2 (0.7)	293	116 (35.8)	324
Gender																
Male	120 (48.2)	249	17 (6.9)	247	9 (3.6)	249	17 (7.7)	220	29 (14.8)	196	46 (19.1)*	241	4 (2.0)	205	47 (20.4)*	230
Female	215 (42.1)	511	41 (8.3)	495	16 (3.1)	508	45 (10.0)	449	57 (15.5)	368	156 (31.5)	495	9 (2.1)	420	177 (38.1)	464
Marital status																
Married	93 (40.8)	228	15 (6.7)	224	13 (5.6)	232	13 (6.2)	210	21 (11.3)	186	54 (24.4)	221	7 (3.7)	187	68 (31.9)	213
Widowed	167 (46.0)	363	31 (8.7)	355	11 (3.0)	366	40 (12.5)	321	42 (16.4)	256	109 (31.5)	346	3 (1.0)	315	110 (33.0)	333
Divorced	10 (52.6)	19	1 (5.3)	19	0 (0.0)	16	2 (13.3)	15	1 (7.7)	13	7 (38.9)	18	0 (0.0)	16	5 (27.8)	18
Never married	22 (59.5)	37	3 (8.1)	37	0 (0.0)	35	0 (0.0)	30	6 (21.4)	28	12 (33.3)	36	1 (3.6)	28	9 (29.0)	31
Living arrangements																
Alone	239 (45.6)	524	40 (7.8)	510	15 (2.9)	520	45 (10.0)	460	66 (17.6)	375	145 (28.8)	504	6 (1.4)	433	157 (32.7)	480
With partner	95 (40.8)	233	16 (7.0)	229	11 (4.7)	234	15 (7.0)	215	18 (9.4)	191	58 (25.7)	226	5 (2.6)	190	71 (32.6)	218
With others	10 (45.5)	22	4 (18.2)	22	1 (4.2)	24	3 (18.8)	16	2 (11.8)	17	9 (40.9)	22	2 (11.1)	18	8 (47.1)	17
Cancer																
Yes	35 (42.2)	83	1 (1.2)	83	1 (1.2)	82	10 (12.7)	79	8 (12.3)	65	21 (25.6)	82	2 (2.6)	78	31 (38.3)	81
No	281 (42.8)	656	52 (8.1)	645	21 (3.2)	648	56 (9.1)	618	69 (14.2)	486	178 (28.3)	630	12 (2.1)	563	204 (32.2)	634
Congestive heart failure																
Yes	73 (62.4)*	117	11 (9.6)	115	3 (2.7)	113	11 (10.6)	104	9 (10.3)	87	29 (27.4)	106	3 (3.1)	96	45 (41.7)	108
No	245 (39.1)	626	42 (6.8)	617	18 (2.9)	618	55 (9.3)	590	68 (14.6)	467	170 (27.8)	611	11 (2.0)	544	190 (31.2)	609
Coronary heart disease																
Yes	50 (50.5)	99	8 (8.2)	98	1 (1.0)	98	4 (4.5)	88	11 (14.7)	75	31 (33.0)	94	1 (1.2)	84	29 (32.2)	90
No	268 (41.7)	643	45 (7.1)	633	20 (3.2)	634	62 (10.2)	607	66 (13.8)	478	168 (27.1)	621	13 (2.3)	556	206 (32.9)	626
COPD[‡]																
Yes	73 (83.9)*	87	5 (5.8)	86	3 (3.4)	87	8 (9.9)	81	11 (16.4)	67	19 (23.5)	81	1 (1.3)	79	28 (34.1)	82
No	244 (37.4)	653	48 (7.5)	643	20 (3.1)	644	58 (9.4)	614	66 (13.6)	484	180 (28.5)	632	13 (2.3)	561	207 (32.6)	634

	CAPs† related to clinical status															
	Cardio-respiratory conditions		Dehydration		Delirium		Faecal incontinence		Nutrition		Pain		Pressure ulcer		Urinary incontinence	
	n (%)	N	n (%)	N	n (%)	N	n (%)	N	n (%)	N	n (%)	N	n (%)	N	n (%)	N
**Total sample**	**350 (42.5)**	**794**	**60 (7.3)**	**775**	**29 (3.5)**	**793**	**66 (8.0)**	**701**	**89 (10.8)**	**591**	**214 (26.0)**	**766**	**14 (1.7)**	**651**	**239 (29.0)**	**725**
Dementia																
Yes	29 (36.3)	80	8 (10.1)	79	13 (16.5)*	79	9 (12.0)	75	10 (15.9)	63	17 (22.1)	77	3 (4.7)	64	23 (30.7)	75
No	290 (43.8)	662	45 (6.9)	652	10 (1.5)	653	57 (9.2)	622	69 (14.1)	489	183 (28.7)	637	11 (1.9)	577	215 (33.4)	644
Diabetes																
Yes	82 (44.1)	186	10 (5.5)	181	5 (2.7)	184	17 (10.3)	165	14 (9.3)	151	58 (32.2)	180	3 (2.0)	152	57 (33.5)	170
No	239 (42.5)	562	45 (8.1)	555	17 (3.1)	553	49 (9.2)	532	64 (15.8)	406	143 (26.5)	540	11 (2.2)	489	179 (32.7)	548
Stroke																
Yes	39 (41.9)	93	8 (8.7)	92	2 (2.2)	90	7 (8.5)	82	7 (10.3)	68	23 (25.3)	91	3 (3.6)	83	35 (41.2)	85
No	281 (43.1)	652	46 (7.2)	642	19 (2.9)	645	59 (9.6)	616	72 (14.8)	486	176 (28.1)	627	11 (2.0)	559	202 (31.9)	634

Abbreviations: † Client Assessment Protocols; ‡ Chronic Obstructive Pulmonary Disorder

Note: CAPs stand for Client Assessment Protocols, which are validated algorithms that alert the assessor to specific problems and risks that can be addressed.

n = number of cases with triggered CAP in the (sub)sample (Note: numbers in subsamples may not add up to the number found in the total sample, due to missing values in subsamples.

N = (sub)sample size

% = prevalence of triggered CAP in (sub)sample (prevalence = n/N)

* = statically significant difference between groups ($p < 0.001$)

Table A2. Absolute number and prevalence of triggered CAPs related to 'Functioning', in total sample and stratified in subgroups (legend below the table).

	CAPs[†] related to functioning													
	ADL		Cognition		Communication		Falls		IADL		Mood		Risk of institutionalization	
	n (%)	N	n (%)	N	n (%)	N	n (%)	N	n (%)	N	n (%)	N	n (%)	N
**Total sample**	**99 (12.0)**	**713**	**13 (1.6)**	**729**	**82 (10.0)**	**804**	**164 (19.9)**	**800**	**293 (35.6)**	**663**	**303 (36.8)**	**803**	**138 (16.7)**	**736**
Age														
65-74	8 (19.0)	42	2 (4.5)	44	12 (22.2)	54	14 (27.5)	51	13 (31.0)	42	29 (54.7)*	53	15 (35.7)	42
75-84	41 (11.7)	350	7 (2.0)	357	35 (8.9)	395	84 (21.5)	391	136 (41.3)	329	169 (42.9)	394	62 (16.8)	370
85+	50 (15.6)	321	4 (1.2)	328	35 (9.9)	355	66 (18.4)	358	144 (49.3)	292	105 (29.5)	356	61 (18.8)	324
Gender														
Male	27 (12.2)	221	4 (1.8)	228	27 (10.8)	251	65 (26.2)	248	84 (40.4)	208	96 (38.7)	248	45 (19.4)	232
Female	67 (14.5)	461	8 (1.7)	468	46 (8.9)	517	91 (17.6)	518	192 (45.3)	424	192 (36.9)	520	81 (17.2)	471
Marital status														
Married	38 (18.1)	210	5 (2.4)	211	28 (12.2)	229	52 (22.8)	228	82 (43.9)	187	96 (41.9)	229	50 (23.8)	210
Widowed	39 (11.9)	329	7 (2.1)	339	30 (8.1)	371	76 (20.7)	367	134 (43.4)	309	130 (35.3)	368	57 (16.7)	342
Divorced	0 (0.0)	16	0 (0.0)	15	1 (5.3)	19	3 (15.8)	19	5 (31.3)	16	10 (52.6)	19	2 (11.8)	17
Never married	4 (12.9)	31	0 (0.0)	33	6 (16.7)	36	6 (16.2)	37	15 (46.9)	32	10 (27.0)	37	6 (17.1)	35
Living arrangements														
Alone	55 (11.7)	472	7 (1.5)	482	48 (9.0)	531	102 (19.3)	529	186 (42.1)	442	189 (35.6)	531	83 (16.8)	493
With partner	33 (15.3)	216	6 (2.8)	215	26 (11.3)	231	54 (23.2)	233	91 (47.2)	193	91 (39.2)	232	44 (20.6)	214
With others	7 (43.8)	16	0 (0.0)	22	2 (8.7)	23	5 (22.7)	22	10 (55.6)	18	13 (59.1)	22	9 (45.0)	20
Cancer														
Yes	9 (11.4)	79	0 (0.0)	79	10 (11.9)	84	16 (19.0)	84	33 (44.0)	75	31 (36.9)	84	14 (17.1)	82
No	89 (14.3)	624	12 (2.0)	608	63 (9.7)	651	134 (20.4)	658	235 (43.0)	546	242 (36.8)	657	113 (18.6)	609
Congestive heart failure	23 (22.1)	104	3 (2.8)	107	12 (10.3)	117	27 (23.1)	117	49 (51.6)	95	49 (41.5)	118	27 (24.1)	112
Yes	74 (12.3)	601	9 (1.5)	583	60 (9.6)	623	124 (19.7)	628	218 (41.1)	530	221 (35.4)	625	100 (17.2)	581
No														
Coronary heart disease	17 (18.7)	91	0 (0.0)	93	8 (8.1)	99	27 (26.7)	101	41 (47.1)	87	27 (27.0)	100	15 (16.1)	93
Yes	81 (13.2)	613	12 (2.0)	597	63 (9.8)	640	125 (19.4)	644	228 (42.5)	537	245 (38.0)	644	112 (18.7)	600
No														
COPD[‡]														
Yes	13 (16.0)	81	0 (0.0)	81	5 (5.8)	86	19 (21.6)	88	25 (35.2)	71	37 (42.0)	88	11 (13.4)	82
No	85 (13.6)	623	12 (2.0)	606	66 (10.1)	652	132 (20.1)	656	240 (43.6)	551	236 (36.0)	655	114 (18.7)	609
Dementia														
Yes	23 (30.7)*	75	9 (11.7)*	77	24 (30.0)*	80	22 (27.5)	80	38 (54.3)	70	41 (51.9)	79	47 (61.8)*	76
No	75 (11.9)	631	4 (0.7)	608	47 (7.1)	659	130 (19.6)	664	232 (42.1)	551	232 (34.9)	664	81 (13.2)	615

Diabetes														
Yes	26 (15.8)	165	2 (1.2)	170	13 (7.0)	185	39 (20.5)	190	72 (46.5)	155	77 (41.0)	188	32 (18.8)	170
No	72 (13.3)	540	10 (1.9)	523	60 (10.7)	561	114 (20.2)	563	202 (42.6)	474	199 (35.4)	562	97 (18.4)	528
Stroke														
Yes	22 (26.5)*	83	1 (1.1)	87	10 (10.8)	93	20 (21.5)	93	36 (43.9)	82	43 (46.7)	92	22 (25.3)	87
No	77 (12.3)	624	11 (1.8)	603	63 (9.7)	648	131 (20.0)	654	234 (43.1)	543	232 (35.5)	653	107 (17.6)	607

Abbreviations: † Client Assessment Protocols; ‡ Chronic Obstructive Pulmonary Disorder

Note: CAPs stand for Client Assessment Protocols, which are validated algorithms that alert the assessor to specific problems and risks that can be addressed.

n = number of cases with triggered CAP in the (sub)sample (Note: numbers in subsamples may not add up to the number found in the total sample, due to missing values in subsamples.

N = (sub)sample size

% = prevalence of triggered CAP in (sub)sample (prevalence = n/N)

* = statically significant difference between groups ($p < 0.001$)

Table A3. Absolute number and prevalence of triggered CAPs related to 'Lifestyle and behaviour', in total sample and stratified in subgroups (legend below the table).

	CAPs† related to lifestyle and behaviour					
	**Behaviour**		**Physical activity**		**Smoking and drinking**	
	**n (%)**	**N**	**n (%)**	**N**	**n (%)**	**N**
**Total sample**	**14 (1.7)**	**804**	**235 (28.5)**	**779**	**129 (15.7)**	**768**
Age						51
65-74	2 (3.8)	53	19 (39.6)	48	11 (21.6)*	378
75-84	6 (1.5)	396	96 (25.1)	382	84 (22.2)	339
85+	6 (1.7)	355	120 (34.4)	349	34 (10.0)	
Gender						
Male	5 (2.0)	250	74 (30.5)	243	54 (22.7)	238
Female	6 (1.5)	517	144 (28.7)	501	67 (13.5)	498
Marital status						
Married	5 (2.2)	230	67 (30.0)	223	41 (18.4)	223
Widowed	3 (0.8)	369	103 (28.6)	360	46 (13.2)	349
Divorced	0 (0.0)	18	6 (33.3)	18	4 (25.0)	16
Never married	0 (0.0)	36	14 (38.9)	36	7 (18.9)	37
Living arrangements						
Alone	8 (1.5)	530	156 (30.3)	515	84 (16.7)	504
With partner	4 (1.7)	233	68 (29.8)	228	37 (16.3)	227
With others	1 (4.3)	23	9 (40.9)	22	6 (27.3)	22
Cancer						
Yes	3 (3.6)	84	18 (22.0)	82	14 (17.3)	81
No	7 (1.1)	654	202 (31.5)	642	106 (16.9)	629
Congestive heart failure						
Yes	3 (2.6)	117	51 (44.7)*	114	14 (12.5)	112
No	7 (1.1)	626	169 (27.4)	616	108 (17.9)	603
Coronary heart disease						
Yes	1 (1.0)	100	28 (28.3)	99	9 (9.6)	94
No	9 (1.4)	643	194 (30.8)	630	111 (17.9)	619
COPD‡						
Yes	1 (1.1)	88	31 (36.0)	86	26 (31.0)*	84
No	9 (1.4)	653	187 (29.2)	641	93 (14.8)	627
Dementia						
Yes	5 (6.3)*	80	22 (27.8)	79	14 (17.5)	80
No	5 (0.8)	661	197 (30.4)	647	110 (17.4)	632
Diabetes						
Yes	2 (1.1)	189	60 (33.1)	181	24 (13.3)	180
No	9 (1.6)	560	164 (29.7)	553	97 (18.0)	540
Stroke						
Yes	1 (1.1)	93	30 (33.7)	89	14 (15.9)	88
No	9 (1.4)	650	192 (30.0)	640	108 (17.2)	628

Abbreviations: † Client Assessment Protocols; ‡ Chronic Obstructive Pulmonary Disorder

Note: CAPs stand for Client Assessment Protocols, which are validated algorithms that alert the assessor to specific problems and risks that can be addressed.

n = number of cases with triggered CAP in the (sub)sample (Note: numbers in subsamples may not add up to the number found in the total sample, due to missing values in subsamples

N = (sub)sample size

% = prevalence of triggered CAP in (sub)sample (prevalence = n/N)

* = statically significant difference between groups ($p < 0.001$)

Table S4. Absolute number and prevalence of triggered CAPs related to 'Social and physical environment', in total sample and stratified in subgroups (legend below the table).

	CAPs† related to social and physical environment							
	**Abusive relationship**		**Home environment**		**Informal care**		**Social functioning**	
	**n (%)**	**N**	**n (%)**	**N**	**n (%)**	**N**	**n (%)**	**N**
**Total sample**	**5 (0.6)**	**410**	**5 (0.6)**	**629**	**376 (45.6)**	**679**	**164 (19.9)**	**778**
Age								
65-74	0 (0.0)	24	3 (7.1)*	42	13 (31.0)*	42	13 (27.7)	47
75-84	2 (1.0)	208	1 (0.3)	310	162 (48.4)	335	87 (22.7)	384
85+	3 (1.7)	178	1 (0.4)	277	201 (66.6)	302	64 (18.4)	347
Gender								
Male	0 (0.0)	124	2 (1.0)	198	86 (40.4)*	213	48 (19.7)	244
Female	3 (1.1)	267	3 (0.7)	406	275 (63.2)	435	111 (22.2)	499
Marital status								
Married	3 (2.3)	129	0 (0.0)	184	8 (4.1)*	193	22 (9.9)*	222
Widowed	1 (0.5)	187	3 (1.0)	290	253 (80.6)	315	99 (27.4)	361
Divorced	0 (0.0)	9	0 (0.0)	14	12 (70.6)	17	4 (22.2)	18
Never married	0 (0.0)	15	1 (3.2)	31	26 (76.5)	34	9 (25.7)	35
Living arrangements								
Alone	3 (1.1)	261	3 (0.7)*	411	376 (81.6)*	461	139 (26.9)*	516
With partner	2 (1.4)	138	0 (0.0)	190	0 (0.0)	200	19 (8.4)	226
With others	0 (0.0)	8	2 (11.8)	17	0 (0.0)	18	2 (8.7)	23
Cancer								
Yes	0 (0.0)	51	2 (2.7)	73	38 (50.0)	76	19 (22.9)	83
No	5 (1.4)	349	3 (0.6)	521	311 (55.7)	558	127 (19.8)	642
Congestive heart failure								
Yes	1 (1.7)	59	1 (1.1)	88	53 (55.2)	96	26 (22.6)	115
No	4 (1.2)	340	4 (0.8)	509	299 (55.3)	541	121 (19.7)	614
Coronary heart disease								
Yes	0 (0.0)	51	0 (0.0)	79	52 (59.1)	88	18 (18.6)	97
No	5 (1.4)	349	5 (1.0)	517	300 (54.6)	549	129 (20.3)	634
COPD‡								
Yes	0 (0.0)	47	1 (1.6)	64	41 (56.9)	72	24 (27.9)	86
No	5 (1.4)	352	4 (0.8)	529	307 (54.5)	563	121 (18.8)	643
Dementia								
Yes	1 (2.0)	49	0 (0.0)	68	23 (33.3)*	69	19 (23.8)	80
No	4 (1.1)	353	5 (1.0)	525	329 (58.1)	566	127 (19.6)	648
Diabetes								
Yes	0 (0.0)	107	2 (1.4)	148	85 (52.8)	161	41 (22.5)	182
No	5 (1.7)	293	3 (0.7)	452	268 (55.8)	480	107 (19.4)	552
Stroke								
Yes	1 (2.0)	51	1 (1.3)	75	44 (51.8)	85	16 (17.2)	93
No	4 (1.1)	351	4 (0.8)	521	309 (55.9)	553	130 (20.3)	639

Abbreviations: † Client Assessment Protocols; ‡ Chronic Obstructive Pulmonary Disorder
Note: CAPs stand for Client Assessment Protocols, which are validated algorithms that alert the assessor to specific problems and risks that can be addressed.
n = number of cases with triggered CAP in the (sub)sample (Note: numbers in subsamples may not add up to the number found in the total sample, due to missing values in subsamples
N = (sub)sample size
% = prevalence of triggered CAP in (sub)sample (prevalence = n/N)
* = statically significant difference between groups ($p < 0.001$)
